# Supplementary material for: Triglyceride-Catabolizing Lactiplantibacillus plantarum GBCC_F0227 Shows an Anti-Obesity Effect in a High-Fat-Diet-Induced C57BL/6 Mouse Obesity Model
Source: Microorganisms. 2024 May 27;12(6):1086. doi: 10.3390/microorganisms12061086 (PMC11205564; doi:10.3390/microorganisms12061086)
Supplement: Supplementary file 1 [file microorganisms-12-01086-s001.zip › microorganisms-2978836-supplementary.pdf]

Supplementary Table S1. Primer sequences used for RT-qPCR analysis

A. Mouse eWAT gene expression

Target gene (Mouse)	Primer sequence (5' - 3')	Product size (bp)
Gapdh	F: TCACCACCATGGAGAAGGC	169
	R: GCTAAGCAGTTGGTGGTGCA	
Adiponectin	F: AAGAAGGACAAGGCCGTTCTCTT	224
	R: GCTATGGGTAGTTGCAGTCAGTT	

B. Bacteria lipase expression

Target gene (Bacteria)	Primer sequence (5' - 3')	Product size (bp)
DNA-directed RNA polymerase subunit beta (rpoβ)	F: CACCGTACCCGTAGAAGTTATGC	106
	R: GGAGACCTTGATCCAAGAACCA	
Proline iminopeptidase (abH11_1)	F: TGCCTACCTGACTGCTAATC	218
	R: CACTGGTGACTAAAGTTGGC	
α/β hydrolase (abH08)	F: AGGCTTACGTATGAGTCGTC	155
	R: CGATCAGGGCCTTAATTCCT	
α/β fold hydrolase (abH08)	F: CTGGTAATCGTAGTGACGTT	168
	R: TCGTAACCTCTGTCGTAGTA	
Proline iminopeptidase (abH11_2)	F: AACCCGACTACTCAGATCCT	170
	R: GTGTTGACCATACTTGAGCG	
Moraxella lipase 2 like (abH04)	F: CACTCATCTCACCGCTACAC	141
	R: TAAACGTGGTCGCAGTATCG	
